# Molecular docking analysis of flavonoids with aldose reductase

**DOI:** 10.6026/97320630018180

**Published:** 2022-03-31

**Authors:** Kasthuri Kannayiram, Vidhya Rekha Umapathy, Y Chamundeswari, JH Fathima, Ramajayam Govindan, Chella Perumal Palanisamy, Vishnu Priya Veeraraghavan, Selvaraj Jayaraman, Ponnulakshmi Rajagopal

**Affiliations:** 1 Department of Biochemistry, Tagore Medical College and Hospital, Melakottaiyur, Chennai, India; 2 Department of Public HealthDentistry, Sree Balaji Dental College and Hospital, Pallikaranai, Chennai-600 100, India; 3 Department of Biochemistry, Allied Health science, Dr. M.G.R Educational and Research Institute, Chennai India; 4 Department of Oral and Maxillofacial Pathology, Ragas Dental College and Hospitals, Chennai, India; 5 Multi Disciplinary Research Unit, Madurai Medical College, TamilNadu, India; 6 State Key Laboratory of Bio-based Materials and Green Papermaking, College of Food Science and Engineering, Qilu University of Technology, Shandong Academy of Science, Jinan 250353, China; 7 Department of Biochemistry, Saveetha Dental College and Hospitals, Saveetha Institute of Medical and Technical Sciences, Saveetha University, Chennai, India

**Keywords:** Diabetes, molecular docking, aldose reductase, flavonoids

## Abstract

Diabetes mellitus is a group of metabolic disorders that has risen to become the third most common cause in humans in recent years. The development of new bioactive substances from natural sources is a relatively new area. Flavonoids are believed to have a variety of beneficial properties in nature, including anti-inflammatory, antimicrobial, anticancer, antioxidant, neuroprotective, and anti-HIV properties. 15 naturally occurring flavonoids docked with the selected target aldose reductase. We report the optimal binding of Acumitin, Agathisflavone, Agehoustin B, and alpha-Toxicarol with aldose reductase for further consideration in drug discovery for T2DM.

## Background:

The global prevalence of diabetes is constantly rising, reaching approximately 382 million in 2013 and possibly reaching 591 million by 2035 [1]. Molecular docking is an effective method for discovering new small molecule drugs that target proteins [2]. Aldose reductase is a member of the super family of Aldo-keto reductases. This is the first rate-limiting enzyme in the polyol pathway, converting glucose to sorbitol with the help of NADPH as a cofactor. The enzyme sorbitol dehydrogenase catalyzes the conversion of sorbitol to fructose [3]. The polyol pathway is a relatively minor mode of glucose utilization, responsible for less than 3% of total glucose utilization. However, when blood glucose levels are elevated, this pathway becomes more active and can account for up to 30% of total glucose intake [4]. During diabetes, abnormal activation of the polyol pathway results in an increase in osmotically active sorbitol, which causes osmotic and oxidative stress, resulting in tissue damage [5]. Though, inhibiting aldose reductase is a fundamental approach to preventing and treating diabetic complications and a possible target for drug development [6]. The aim of this study is to identify potential lead drug molecules from flavonoids that inhibit aldose reductase. Flavonoids are synthesized through the phenylpropanoid pathway, which converts phenylalanine to 4-coumaroyl-CoA, which would then be transferred to the flavonoid pathway [7]. They exhibit a broad spectrum of biological activities, including antioxidant [8], anticancer [9], antibacterial [10], antifungal [11], and antiviral [12]. Therefore, it is of interest to document the Molecular docking analysis of flavonoids with aldose reductase.

## Materials and Methods:

### Ligand preparation:

15 Flavonoids compounds were chosen from the National NPACT natural compound database (Table 1 - see PDF). These molecules were downloaded in Structure Date File (SDF) format and converted to Protein Data Bank (PDB) coordinates by using Open Babel (http://openbabel.org) converter.

### Protein preparation:

The structure of aldose reductase complexed with peptide substrate was obtained from PDB data bank (PDB Code: 1IEI). The resolution factor is 1.45Å and the method of incorporation is X-ray diffraction method. The receptors were minimized using the Swiss PDB viewer, and the active site residue was identified. The ligand molecules associated to the target have been picked using the control panel of this stand-alone program. All residues surrounding the ligand have been identified and picked. The ligand molecules were separated using Argus lab, and the final preparation was accomplished by removing water molecules and adding H-atoms.

### Molecular docking:

The molecular docking analyses were carried using the PyRx docking method through AutoDock VINA programme to determine the potential mode of interaction between selected phytocompounds with aldose reductase protein [13, 14]. In the PyRx tool, the macromolecule was uploaded, which immediately eliminates the solvent molecules followed by measurements of hydrogen addition and gasteiger charges. In the PyRx tool associated with AutoDock VINA, the small molecules were uploaded. The receptor and compounds were translated into a pdbqt format. On the active sites of the protein, the Grid centre was located. To optimize the binding conformational analysis, the default exhaustively value has been used. Based on binding affinity values (kcal/mol) and bonding interaction patterns, the produced docked complexes were examined (hydrogen, hydrophobic, and electrostatic). The docked complexes were graphically described by PyMOL version 1.7.2 (PyMOL Molecular Graphics System) programme (DeLano Scientific LLC, San Carlos, CA, USA).

## Results and Discussion:

The conventional therapeutic method offers several possibilities for the treatment of several illnesses that have yet to be explored. If modern computational chemistry methods are used to investigate the ability of the conventional medicinal method, astounding results can be obtained. Numerous scientists have taken away similar experiments in the past in which bioactive compounds are docked on a specific receptor to determine its affinity. The docking poses in this study were rated according to their docking ratings. The best four compound complexes (Acumitin, Agathisflavone, Agehoustin B, and alpha-Toxicarol) were chosen based on these scoring parameters (Table 2 - see PDF). The docked positions of selected compounds with aldose reductase confirmed the ligand's binding positions with the enzyme. If a compound has a lower binding energy in docking experiments, this indicates that the compound could have a higher activity. The four compounds chosen had the lowest binding energies with the target protein. The hydrogen bond also plays a critical role in the inhibitory action of the target protein. All four compounds developed several hydrogen bond interactions with the target protein, indicating that they have the ability to inhibit aldose reductase activity. PHE-161, GLN-192, ARG-296, ASN-182, ARG-225, CYS-298, and ALA-299 form hydrogen bond interactions with AR in an alternate fashion (Figure 1 - see PDF).

Additionally, the Insilco findings demonstrated that lead molecules can substantially reduce intracellular sorbitol accumulation, which has been implicated in the pathogenesis of late-onset diabetic complications such as retinopathy, neuropathy, and nephropathy. The majority of synthetic Aldose reductase (AR) inhibitors have been removed from the market due to adverse side effects and suboptimal pharmacokinetic properties. Now, we're looking for a safe and efficient lead molecule to help us develop more effective AR inhibitors. Numerous reports exist in the literature describing the inhibitory action of various phytochemicals derived from historically essential medicinal plants on aldose reductase. Thus, compounds discovered via this process can also serve as a potential AR inhibitor after experimental verification.

## Conclusion:

We report the optimal binding of Acumitin, Agathisflavone, Agehoustin B, and alpha-Toxicarol with aldose reductase for further consideration in drug discovery for T2DM.
